# Association between new indices in the locomotive syndrome risk test and decline in mobility: third survey of the ROAD study

**DOI:** 10.1007/s00776-015-0741-5

**Published:** 2015-06-25

**Authors:** Noriko Yoshimura, Shigeyuki Muraki, Hiroyuki Oka, Sakae Tanaka, Toru Ogata, Hiroshi Kawaguchi, Toru Akune, Kozo Nakamura

**Affiliations:** Department of Joint Disease Research, 22nd Century Medical and Research Center, The University of Tokyo, Hongo 7-3-1, Bunkyo-ku, Tokyo, 113-8655 Japan; Department of Clinical Motor System Medicine, 22nd Century Medical and Research Center, The University of Tokyo, Tokyo, 113-8655 Japan; Department of Medical Research and Management for Musculoskeletal Pain, 22nd Century Medical and Research Center, The University of Tokyo, Tokyo, 113-8655 Japan; Department of Orthopaedic Surgery, Sensory and Motor System Medicine, Graduate School of Medicine, The University of Tokyo, Tokyo, 113-8655 Japan; National Rehabilitation Center for Persons with Disabilities, Saitama, 359-0042 Japan; JCHO Tokyo Shinjuku Medical Center, Tokyo, 162-8542 Japan

## Abstract

**Background:**

We aimed to clarify the association between new indices in a locomotive syndrome risk test and decline in mobility.

**Methods:**

In the third survey of the Research on Osteoarthritis/osteoporosis Against Disability (ROAD) study, data on the indices were obtained from 1575 subjects (513 men, 1062 women) of the 1721 participants in mountainous and coastal areas. As outcome measures for decline in mobility, we used the five-times-sit-to-stand test (FTSST) and walking speed with cutoff values of 12 s and 0.8 m/s, respectively.

**Results:**

We first estimated the prevalence of the indices in locomotive syndrome risk test stage 1, including two-step test score <1.3, difficulty with one-leg standing from a 40-cm-high seat in the stand-up test, and 25-question GLFS score ≥7, which were found to be 57.4, 40.6, and 22.6 %, respectively. Next, we investigated the prevalence of the indices in locomotive syndrome risk test stage 2, including two-step test score <1.1, difficulty with standing from a 20-cm-high seat using both legs in the stand-up test, and 25-question GLFS score ≥16, which were found to be 21.1, 7.9, and 10.6 %, respectively. Logistic regression analysis using slow FTSST time or slow walking speed as the objective factor, and presence or absence of indices as the independent factor, after adjusting for confounders, showed all three indices in both stages 1 and 2 were significantly and independently associated with immobility. Finally, we clarified the risk of immobility according to an increasing number of indices in both stages 1 and 2 and found that the odds ratio for both slow FTSST time and slow walking speed increased exponentially.

**Conclusion:**

We found that the three indices independently predicted immobility and that accumulation of indices increased the risk of immobility exponentially.

**Electronic supplementary material:**

The online version of this article (doi:10.1007/s00776-015-0741-5) contains supplementary material, which is available to authorized users.

## Introduction

According to the most recent National Livelihood Survey by the Ministry of Health, Labour, and Welfare in Japan, osteoporotic fracture and falls is ranked fourth and osteoarthritis is ranked fifth among conditions that cause disability and subsequently require support with regard to activities of daily living [1]. Given the increasing proportion of elderly individuals in the Japanese population, a comprehensive and evidence-based prevention strategy for musculoskeletal diseases is urgently required. In 2007, the Japanese Orthopaedic Association (JOA) proposed that the term “locomotive syndrome” should be adopted to designate a condition requiring nursing care, or being at risk of developing such a condition, because of a decline in mobility resulting from a disorder of the locomotive system, which consists of bones, joints, muscles, and nerves [2]. Weakness of such locomotive components causes difficulty in mobility—defined as the ability to stand, walk, run, climb stairs, and perform other physical functions essential to daily life.

As candidate indices to assess the risk of locomotive syndrome, in 2013, the JOA proposed the following three tests: two-step test, stand-up test, and 25-question geriatric locomotive function scale (GLFS) [3]. With regard to the stand-up test, more than 50 % of subjects younger than 70 years old can stand up on one leg from a 40-cm-high seat [3]. The 25-question GLFS has already been assessed regarding its sensitivity and specificity for prediction of disability and was assigned a cutoff value of 16 by Seichi et al. [4]. However, there is little information regarding reference and/or cutoff values for the two-step test.

Recently, the JOA determined clinical decision limits of these three indices for assessing risk of locomotive syndrome [5]. In their proposal, clinical decision limits were established in two stages as follows:

- Stage 1:

1. Two-step test score <1.3.
2. Difficulty with one-leg standing from a 40-cm-high seat in the stand-up test (either leg).
3. 25-question GLFS score ≥7.

When a subject meets any of the above-mentioned conditions, he/she is diagnosed as starting to decline in mobility.

- Stage 2:

1. Two-step test score <1.1.
2. Difficulty with standing from a 20-cm-high seat using both legs in the stand-up test.
3. A 25-question GLFS score ≥16.

When a subject meets any of the above-mentioned conditions, he/she is diagnosed as progressing to a decline in mobility.

However, no report has evaluated such indices using data of the general population. From 2005 to 2007, we started a large-scale, population-based cohort investigation entitled the Research on Osteoarthritis/osteoporosis Against Disability (ROAD) study, consisting of 3040 participants in three communities located in urban, mountainous, and coastal areas. Following the baseline study, we performed a second survey in the same communities from October 2008 to January 2010, followed by a third survey from October 2012 to December 2013. In the third survey, participants completed the two-step test, stand-up test, and 25-question GLFS. In the present report, using data from the third survey of the ROAD study, we assessed the usefulness of these new indices for predicting immobility, which causes subsequent disability.

## Participants and methods

### Participants

Measurements were obtained from participants of the third survey of the ROAD study. The ROAD study, which began in 2005, is a nationwide prospective study comprising population-based cohorts established in several communities in Japan. Recruitment methods for this study have been described in detail elsewhere [6, 7]. To date, we have created a baseline database including clinical and genetic information of 3040 inhabitants (1061 men; 1979 women) aged 23–95 years who were recruited from listings of resident registrations in three communities. All participants provided written informed consent, and the study was conducted with approval from the ethics committees of the participating institutions.

The third survey of the ROAD study began in 2012 and was completed in 2013. All participants in the baseline study and second survey were invited to participate in the third survey. Besides former participants, inhabitants aged ≥40 years who were willing to attend the ROAD survey performed in 2012–2013 also were included as participants in the third survey. As a result, a total of 2566 (837 men, 1729 women; urban area, 845 individuals; mountainous area, 769 individuals; coastal area, 952 individuals) residents participated in the third survey.

In the present study, we used data from 1575 subjects (513 men; 1062 women) who completed the stand-up test, two-step test, and 25-question GLFS for disability among all 1721 participants in mountainous and coastal areas in the third survey.

At the third survey, participants completed an interviewer-administered questionnaire. Five interviewers, who had been trained by an expert (NY), were provided for this study. The questionnaire consisted of 200 items that included lifestyle information, such as primary occupation, smoking habits, alcohol consumption, physical activity, medical history, and prescription medication. Anthropometric measurements included height (cm), weight (kg), body mass index [BMI, weight (kg)/height (m)^2^], and hand grip strength (kg).

### Indices for risk of decline in mobility resulting from locomotive syndrome

In the present study, participants performed the following tests for assessment of decline in mobility.

#### Two-step test

This test measures the stride length to assess walking ability, including muscle strength, balance, and flexibility of the lower limbs. The two-step test was performed using the following procedure [3, 8, 9]: (1) subjects determined the starting line and stood with the toes of both feet behind it; (2) subjects were instructed to take two long steps (as long as possible) and then align both feet; (3) the length of the two steps from the starting line to the tips of the subject’s toes where he/she stopped was measured. The two-step test score was calculated using the following formula: length of the two steps (cm) ÷ height (cm).

#### Stand-up test

This test assesses leg strength by having the subject stand up on one or both legs from a specified height. After preparation of four seats of different heights—40, 30, 20, and 10 cm—the subject stood up from each seat (in descending height order), first with both legs then with one leg. If the subject could stand up without leaning back to gain momentum and maintain the posture for 3 s, then he/she was diagnosed as having passed that height level [3, 9]. In the present study, if the subject was unable to stand up on one leg (right or left) from a height of 40 cm, then his/her stand-up test was considered failed.

#### The 25-Question GLFS

As mentioned above, the 25-question GLFS was developed by Seichi et al. [4]. It is a self-administered, comprehensive measure, consisting of 25 items that include four questions regarding pain during the last month, 16 questions regarding activities of daily living during the last month, three questions regarding social functions, and two questions regarding mental health status during the last month. These 25 items are graded with a five-point scale, from no impairment (0 points) to severe impairment (4 points), and then arithmetically added to produce a total score (minimum = 0, maximum = 100). Thus, a higher score is associated with worse locomotive function. Validity of the scale has been assessed, and a cutoff point of 16 was determined to have the highest sensitivity and specificity for indication of disability resulting from locomotive syndrome [3, 4].

### Indices for decline in mobility resulting from locomotive syndrome

Because the present study utilized a cross-sectional design, not a longitudinal follow-up design, we could not evaluate the ability of the stand-up test or two-step test for prediction of disability resulting from locomotive syndrome. Therefore, in the present study, we used the following outcome measures as indices for decline in mobility.

#### Five-times-sit-to-stand test

There are several reports that inability to rise from a chair five times within a determined time is associated with increased disability and morbidity [10–12]. We have also reported that the longer the standing time is, the higher the incidence of disability [13]. The five-times-sit-to-stand test (FTSST) was performed according to the following procedure: (1) using a straight-back chair with a solid seat, participants were asked to sit on the chair with their arms folded across their chest; (2) participants were instructed to stand up and sit down as quickly as possible five times, keeping their arms folded across their chest; (3) the time when the participant stood for the fifth time was measured. In the present study, we used a cutoff value of 12 s to indicate a decline in mobility [14].

#### The 6-m walking time

As another outcome measure for decline in mobility, participants walked a 6-m course at their usual speed. The method of measurement of walking time was identical to that performed in the large-scale cohort study entitled Osteoporotic Fractures in Men (MrOS), which started prior to the ROAD study [15]. In the present study, we used a cutoff value of 0.8 m/s to indicate a decline in mobility [16].

In the present study, among the above-mentioned indices, mean scores and SDs for the two-step test were calculated according to participants’ sex and age strata (<40, 40s, 50s, 60s, 70s, and ≥80 years). Then, we estimated the prevalence of each index in stages 1 and 2. Finally, we assessed the association between the cumulative number of indices and decline in mobility using multivariate analysis.

### Statistical analysis

All statistical analyses were performed using STATA statistical software (STATA, College Station, TX, USA). Differences in proportions were compared using the chi-square test. The significance of differences in continuous variables was evaluated using analysis of variance for comparisons among multiple groups or Scheffe’s least significant difference test for pairs of groups. All *p* values and 95 % confidence intervals are two-sided. A *p* value of <0.05 was considered statistically significant.

Logistic regression analysis was used to test the association of each factor with the presence or absence of a decline in mobility. In the analysis, we used presence of immobility according to the FTSST time (>12 s = 1; ≤12 s = 0) and usual walking speed (<0.8 m/s = 1; ≥0.8 m/s = 0) as the objective variable, and presence or absence of new indices in stages 1 and 2 as explanatory variables, after adjusting for age (+1 year), sex (men = 0, women = 1), BMI (+1 kg/m^2^), and regional difference (mountainous area = 0; coastal area = 1). Other factors were considered in the multivariate model after simple linear analysis; those used as explanatory factors are described in “Results.”

## Results

Summary characteristics, including age and BMI of the participants, are shown in Table 1. Two-thirds of the 1575 participants were women, and the mean age of women participants was 1 year less than that of men participants; however, this difference was not significant. By contrast, there was a significant difference in BMI between sexes (*p* < 0.0001). Table 1 also shows the age and sex distributions of mean FTSST time and walking speed. Both values tended to be significantly slower in participants aged in their 70s and 80s in both men and women, and there were no significant differences between sexes. Table 1 also shows the age and sex distributions of mean two-step test scores. Mean two-step test score was 1.25 (SD 0.20) in men and 1.23 (SD 0.21) in women; this difference was significant (*p* < 0.0001). Age differences indicated that the two-step test score was significantly lower according to age in both men and women (*p* < 0.05).

**Table 1:** Mean (SD) values for age, body mass index (BMI), five-times-sit-to-stand test (FTSST) time, walking speed, and two-step test score of participants classified by age and sex

Age strata (years)	*n*	Age (years)	BMI (kg/m^2^)	FTSST time (s)	Walking speed (m/s)	Two-step test score
Men						
<40	23	32.8 (4.8)	24.5 (3.3)	6.96 (1.33)	1.26 (0.22)	1.49 (0.14)
40–49	38	44.7 (3.1)	25.4 (5.1)	6.79 (2.41)	1.25 (0.25)	1.41 (0.15)
50–59	82	55.2 (2.5)	24.2 (3.3)	7.11 (1.47)	1.25 (0.26)	1.36 (0.13)^a^
60–69	137	64.3 (2.7)	23.8 (3.4)	8.10 (2.51)	1.16 (0.26)	1.29 (0.15)^ab^
70–79	139	74.3 (2.8)	23.4 (2.9)	8.72 (2.18)^bc^	1.02 (0.24)^abcd^	1.20 (0.16)^abcd^
≥80	94	83.8 (3.1)	22.3 (3.0)	11.48 (4.72)^abcde^	0.81 (0.28)^abcde^	1.06 (0.22)^abcde^
Total	513	66.2 (13.7)	23.6 (3.4)	8.57 (3.17)	1.08 (0.30)	1.25 (0.20)
Women						
<40	36	34.4 (4.8)	20.7 (3.0)	7.11 (1.26)	1.28 (0.17)	1.40 (0.14)
40–49	85	44.9 (2.9)	21.9 (3.2)	7.19 (1.64)	1.25 (0.22)	1.35 (0.11)
50–59	195	54.7 (2.9)	23.0 (4.1)	7.10 (1.94)	1.26 (0.22)	1.35 (0.13)
60–69	309	64.7 (2.9)	22.8 (3.4)	7.90 (2.31)	1.18 (0.23)^c^	1.28 (0.18)^abc^
70–79	303	74.3 (2.9)	23.3 (3.3)	9.44 (3.57)^abcd^	1.02 (0.28)^abcd^	1.16 (0.18)^abcd^
≥80	134	83.1 (3.0)	22.0 (3.4)	11.89 (4.60)^abcde^	0.75 (0.28)^abcde^	0.97 (0.23)^abcde^
Total	1062	65.3 (12.6)	22.7 (3.5)	8.58 (3.31)	1.11 (0.30)	1.23 (0.21)

^a^Significantly different (*p* < 0.05) from values of those aged <40 years

^b^Significantly different (*p* < 0.05) from values of those aged in their 40s

^c^Significantly different (*p* < 0.05) from values of those aged in their 50s

^d^Significantly different (*p* < 0.05) from values of those aged in their 60s

^e^Significantly different (*p* < 0.05) from values of those aged in their 70s

First, the prevalence of the indices in stage 1 and their association with decline in mobility described by slow FTSST time and slow walking speed were assessed (Table 2). Overall, the prevalence of two-step test score <1.3, difficulty with one-leg standing from a 40-cm-high seat in the stand-up test, and 25-question GLFS score ≥7 were 57.4, 40.6, and 22.6 %, respectively.

**Table 2:** Prevalence of indices in the locomotive syndrome risk test (stage 1): two-step test score <1.3, difficulty with one-leg standing from 40-cm-high seat in the stand-up test (either leg), and 25-question geriatric locomotive function scale (GLFS) score ≥7 in participants classified by age and sex

Age strata (years)	Age (years) mean (SD)	Two-step test score <1.3 (%)	Difficulty with one-leg standing from 40-cm-high seat (either leg) (%)	25-question GLFS score ≥7 (%)
Men				
<40	32.8 (4.8)	13.0	4.4	4.4
40–49	44.7 (3.1)	21.1	15.8	10.8
50–59	55.2 (2.5)	34.6	15.9	7.4
60–69	64.3 (2.7)	49.3	30.7	12.0
70–79	74.3 (2.8)	71.7	47.8	19.9
≥80	83.8 (3.1)	84.6	78.0	44.0
Total	66.2 (13.7)	55.6	39.1	18.8
Women				
<40	34.4 (4.8)	19.4	11.1	0.0
40–49	44.9 (2.9)	31.8	12.9	8.3
50–59	54.7 (2.9)	31.8	23.6	13.0
60–69	64.7 (2.9)	52.4	33.9	19.7
70–79	74.3 (2.9)	78.3	56.2	31.6
≥80	83.1 (3.0)	93.8	78.1	54.3
Total	65.3 (12.6)	58.2	41.3	24.5*

* Significantly different (*p* < 0.05) from values of men

Prevalence of two-step test score <1.3 in subjects aged in their 30s and younger, 40s, 50s, 60s, 70s, and 80 years and older were 17.0, 28.5, 32.6, 51.5, 76.2, and 90.0 %, respectively, indicating that the prevalence increased according to age; even in subjects aged in their 40s and 50s, the prevalence was more than 30 %. Prevalence of subjects who could not stand with one leg from a 40-cm-high seat who were aged in their 30s and younger, 40s, 50s, 60s, 70s, and 80 years and older were 8.5, 13.8, 21.3, 32.9, 53.5, and 78.1 %, respectively, indicating that the prevalence increased according to age, similar to the two-step test; even in subjects aged in their 40s and 50s, the prevalence was around 20 %. Prevalence of 25-question GLFS score ≥7 in participants aged in their 30s and younger, 40s, 50s, 60s, 70s, and 80 years and older were 1.7, 9.1, 11.4, 17.4, 27.9, and 50.0 %, respectively, indicating the overall prevalence was lower than that of the other two indices, but it increased synergistically in those in their 80s and older. Regarding the sex difference of the indices in stage 1, although there were no significant differences between men and women with regard to two-step test score <1.3 and difficulty with one-leg standing from a 40-cm-high seat in the stand-up test, the prevalence of 25-question GLFS score ≥7 in women was significantly higher than that in men (*p* < 0.05).

Table 3 shows the results of logistic regression analysis using immobility described by slow FTSST time or slow walking speed as the objective factor and the presence or absence of new indices in stage 1 for a decline in mobility as explanatory factors, after adjusting for age (+1 year), sex (men = 0; women = 1), BMI (+1 kg/m^2^), and regional difference (mountainous area = 0; coastal area = 1). The analysis revealed that all three indices in stage 1 independently predicted immobility described by both slow FTSST time and slow walking speed.

**Table 3:** Effect of presence of indices in the locomotive syndrome risk test (stage 1) for decline in mobility described by slow five-times-sit-to-stand test (FTSST) time and slow walking speed

Indices for decline in mobility	Reference	OR (95 % CI)	*p* value
FTSST time >12 s			
Two-step test score <1.3	Yes vs. no	3.28 (1.81–5.97)	<0.001
Difficulty with one-leg standing from 40-cm-high seat (either leg)	Yes vs. no	1.78 (1.17–2.69)	0.007
25-question GLFS score ≥7	Yes vs. no	2.51 (1.73–3.62)	<0.001
Walking speed <0.8 m/s			
Two-step test score <1.3	Yes vs. no	4.24 (2.18–8.22)	<0.001
Difficulty with one-leg standing from 40-cm-high seat (either leg)	Yes vs. no	2.01 (1.35–3.16)	0.001
25-question GLFS score ≥7	Yes vs. no	2.65 (1.82–3.86)	<0.001

After adjusting for age, sex, body mass index, and regional difference. Presence or absence of indices for stage 1 also was mutually adjusted

*CI* confidence interval, *GLFS* geriatric locomotive function scale, *OR* odds ratio

Table 4 shows the association between accumulation of the indices in stage 1 and decline in mobility described by slow FTSST time or slow walking speed, after adjusting for age (+1 year), sex (men = 0; women = 1), BMI (+1 kg/m^2^), and regional difference (mountainous area = 0; coastal area = 1). The analysis revealed that according to an increasing number of indices, the odds ratio of both slow FTSST time and slow walking speed increased exponentially.

**Table 4:** Effect of accumulation of indices (stage 1) for decline in mobility

No. of indices	OR (95 % CI)	*p* value
Five-times-sit-to-stand test time >12 s		
0 = reference	1.0	–
1	2.19 (0.98–4.87)	0.055
2	2.90 (1.30–6.47)	0.009
3	11.59 (5.18–25.93)	<0.001
Walking speed <0.8 m/s		
0 = reference	1.0	–
1	5.73 (1.71–19.16)	0.005
2	9.82 (2.96–32.52)	<0.001
3	32.21 (9.64–107.7)	<0.001

After adjusting for age, sex, body mass index, and regional difference

*CI* confidence interval, *OR* odds ratio

Next, the prevalence of the indices in stage 2 and their association with decline in mobility described by a slow FTSST time and slow walking speed were assessed (Table 5). Overall, the prevalence of two-step test score <1.1, difficulty with standing from a 20-cm-high seat using both legs in the stand-up test, and 25-question GLFS score ≥16 were 21.1, 7.9, and 10.6 %, respectively.

**Table 5:** Prevalence of indices in the locomotive syndrome risk test (stage 2): two-step test score <1.1, difficulty with standing from 20-cm-high seat using both legs in the stand-up test, and 25-question geriatric locomotive function scale (GLFS) score ≥16 in participants classified by age and sex

Age strata (years)	Age, mean (SD) years	Two-step test score <1.1 (%)	Difficulty with standing from 20-cm-high seat (%)	25-question GLFS score ≥16 (%)
Men				
<40	32.8 (4.8)	0.0	0.0	0.0
40–49	44.7 (3.1)	2.6	2.6	0.0
50–59	55.2 (2.5)	3.7	0.0	1.2
60–69	64.3 (2.7)	8.8	3.7	6.0
70–79	74.3 (2.8)	23.9	2.9	8.1
≥80	83.8 (3.1)	58.2	16.5	27.5
Total	66.2 (13.7)	20.1	4.9	9.0
Women				
<40	34.4 (4.8)	0.0	0.0	0.0
40–49	44.9 (2.9)	1.2	0.0	2.4
50–59	54.7 (2.9)	3.1	1.0	4.2
60–69	64.7 (2.9)	12.4	5.5	4.6
70–79	74.3 (2.9)	30.4	13.1	15.1
≥80	83.1 (3.0)	71.1	31.3	39.4
Total	65.3 (12.6)	21.6	9.4^*^	11.4

* Significantly different (*p* < 0.01) from values of men

Prevalence of two-step test score <1.1 in subjects aged in their 30s and younger, 40s, 50s, 60s, 70s, and 80 years and older were 0.0, 1.6, 3.3, 11.3, 28.4, and 65.8 %, respectively, indicating that the prevalence was less than 5 % in those aged in their 50s and younger, around 10 % in those aged in their 60s, but more than 50 % in those aged 80 years and older. Prevalence of subjects who could not stand from a 20-cm-high seat using both legs who were aged in their 30 s and younger, 40s, 50s, 60s, 70s, and 80 years and older were 0.0, 0.8, 0.7, 5.0, 9.9, and 25.1 %, respectively, indicating that the prevalence was less than 5 % in those aged in their 60s and younger, around 10 % in those aged in their 70s, but dramatically increased in those aged 80 years and older. Prevalence of 25-question GLFS score ≥16 in participants aged in their 30s and younger, 40s, 50s, 60s, 70s, and 80 years and older were 0.0, 1.7, 3.3, 5.0, 12.9, and 34.4 %, respectively, indicating that the overall prevalence increased according to age; the prevalence was less than 5 % in subjects aged in their 60s and younger, around 10 % in those aged in their 70s, but dramatically increased in those aged 80 years and older. Regarding the sex difference of the indices in stage 2, although there were no significant differences between men and women with regard to two-step test score <1.1 and 25-question GLFS score ≥16, the prevalence of difficulty with standing from a 20-cm-high seat using both legs in the stand-up test in women was significantly higher than that in men (*p* < 0.01).

Table 6 shows the results of logistic regression analysis using immobility described by a slow FTSST time or slow walking speed as the objective factor and the presence or absence of new indices in stage 2 for decline in mobility as explanatory factors, after adjusting for age (+1 year), sex (men = 0; women = 1), BMI (+1 kg/m^2^), and regional difference (mountainous area = 0; coastal area = 1). The analysis revealed that all three indices in stage 2 independently predicted immobility described by both a slow FTSST time and slow walking speed. The odds ratios of all indices in stage 2 for slow FTSST time and slow walking speed were greater than those of all indices in stage 1.

**Table 6:** Effect of presence of indices in the locomotive syndrome risk test (stage 2) for decline in mobility described by slow five-times-sit-to-stand test (FTSST) time and slow walking speed

Indices for decline in mobility	Reference	OR (95 % CI)	*p* value
FTSST time >12 s			
Two-step test score <1.1	Yes vs. no	3.03 (1.97–4.66)	<0.001
Difficulty with standing from 20-cm-high seat %	Yes vs. no	5.87 (3.48–9.89)	<0.001
25-question GLFS score ≥16	Yes vs. no	2.83 (1.77–4.54)	<0.001
Walking speed <0.8 m/s			
Two-step test score <1.1	Yes vs. no	4.19 (2.75–6.39)	<0.001
Difficulty with standing from 20-cm-high seat %	Yes vs. no	3.40 (1.99–5.82)	<0.001
25-question GLFS score ≥16	Yes vs. no	3.49 (2.15–5.65)	<0.001

After adjusting for age, sex, body mass index, and regional difference. Presence or absence of indices for stage 2 also was mutually adjusted

*CI* confidence interval, *GLFS* geriatric locomotive function scale, *OR* odds ratio

Table 7 shows the association between accumulation of the indices in stage 2 and decline in mobility described by slow FTSST time or slow walking speed, after adjusting for age (+1 year), sex (men = 0; women = 1), BMI (+1 kg/m^2^), and regional difference (mountainous area = 0; coastal area = 1). The analysis revealed that according to an increasing number of indices, the odds ratio of both slow FTSST time and slow walking speed increased exponentially. The odds ratios of accumulation of the indices in stage 2 for slow FTSST time and slow walking speed were greater than those of accumulation of the indices in stage 1.

**Table 7:** Effect of accumulation of new indices (stage 2) for decline in mobility

No. of new indices	OR (95 % CI)	*p* value
Five-times-sit-to-stand test time >12 s		
0 = reference	1.0	–
1	2.99 (1.85–4.84)	<0.001
2	12.35 (7.08–21.54)	<0.001
3	46.87 (19.37–113.45)	<0.001
Walking speed <0.8 m/s		
0 = reference	1.0	–
1	3.50 (2.19–5.59)	<0.001
2	12.52 (7.08–22.13)	<0.001
3	61.93 (24.92–153.87)	<0.001

After adjusting for age, sex, body mass index, and regional difference

*CI* confidence interval, *OR* odds ratio

## Discussion

In the present study, we clarified the associations of three new indices for immobility, including the two-step test, stand-up test, and 25-question GLFS, represented by slow FTSST time and slow walking speed. We first tested the associations among the three indices by using the clinical decision limits for locomotive syndrome risk test stage 1. Next, we tested the three indices in stage 2. We clarified the age and sex distributions of the prevalence of these three indices and found that the three indices in both stages 1 and 2 significantly and independently predicted a decline in mobility and that the accumulation of such indices increased the risk of immobility exponentially.

First, we used both FTSST time and 6-m walking speed as outcome measures of immobility. As mentioned in “Methods”, these two indices are both regarded as predictors for morbidity and disability [10–12, 16]. In the ROAD study, we reported that the longer the standing time, the higher the incidence of disability [13] and that slow walking speed was also a predictor for the occurrence of disability [13]. Although we could not clarify the direct associations between these new indices and occurrence of disability because this study design was cross-sectional, these surrogate indices could help predict disability in the near future. Therefore, if we could clarify the significant associations between these new indices and FTSST time and 6-m walking speed, we might clarify the ability to predict disability indirectly. Based on this hypothesis, we used cutoff values of 12 s for FTSST time [13] and 0.8 m/s for 6-m walking speed [16].

The two-step test was developed to assess walking ability, including muscle strength, balance, and flexibility of the lower limbs. This test was first developed by Muranaga and Hirano in 2003 [3, 8]. They compared two-step test scores of 108 healthy volunteers (38 men, 70 women; mean age 59.0 years) with those of 108 hospital outpatients (56 men, 52 women; mean age 60.3 years) and found that the two-step test score was significantly associated with the risk of falling and degree of independence in daily life [7]. In the present study, we clarified mean two-step test scores in participants classified by age and sex and found mean scores of 2.07 in men and 1.87 in women. Scores of men were significantly higher than those of women, and age differences indicated that scores were significantly lower according to age in both men and women. These age and sex tendencies are consistent with those reported in a previous study [3].

Regarding the prevalence of the indices in locomotive syndrome risk test stages 1 and 2, we found that all prevalences increased with age. However, the distribution of each index seemed to differ. In stage 1, the prevalence was highest for a two-step test score <1.3, followed by difficulty with one-leg standing from a 40-cm-high seat in the stand-up test and 25-question GLFS score ≥7. By contrast, in stage 2, the prevalence also was highest for a two-step test score <1.1, but the prevalence of a 25-question GLFS score ≥16 was higher than that of difficulty with standing from a 20-cm-high seat using both legs in the stand-up test. The different age distributions of these three indices in both stages 1 and 2 might be conducive to their mutually independent associations with immobility. Our result that these three indices in both stages 1 and 2 independently predicted immobility shows that all three are important for predicting immobility. Furthermore, because these indices independently predicted immobility, risk severity may be classified by a positive number of indices present. In fact, in our study, accumulation of indices increased the risk of immobility exponentially, especially accumulation of indices in stage 2, which suggests the possibility of categorizing the severity of risk for immobility, such as 0, normal; 1, mild; 2, moderate; 3, severe.

With regard to the 25-question GLFS, there might be some concern that it is too cumbersome for older people to answer 25 questions. Seichi et al. also proposed a short version of the test using only five questions with a cutoff score of ≥6, on behalf of a 25-question GLFS score ≥16. They selected five questions from the 25-question GLFS using the Akaike information criterion (AIC) [17–19]. They determined that a cutoff score of 6 had the lowest AIC value, representing the model with the best fit, and finally concluded that the five-question GLFS can be applied as a rapid self-check tool for locomotive syndrome [4]. We compared the results of 1535 individuals who completed both the 25-question GLFS and five-question GLFS. Supplementary Table 1 shows that these two indices (five-question GLFS and 25-question GLFS) have a strong association (sensitivity 0.859; specificity 0.985). This result shows the possibility of using the five-question GLFS instead of the 25-question GLFS as a rapid-check tool for the prediction of immobility.

There are several limitations in the present study. First, as mentioned above, our study assessed the usefulness of three indices in locomotive syndrome risk test stages 1 and 2 for predicting immobility using FTSST time and walking speed as outcome variables. Although there has been significant evidence regarding these outcome measures, such as slow FTSST time and slow walking speed, and disability [8, 10–13, 16], including our report, the direct associations of these new indices and occurrence of disability are unclear. The proposal of these new indices was published in 2013 [3], so there was not enough time to observe future occurrence of disability in our cohort. Therefore, we should continue to observe our cohort and assess the ability of such indices to predict disability directly. Second, although the ROAD study includes a large number of participants, these participants do not truly represent the general population, since the subjects in the present study were recruited from only two areas. However, we have already confirmed the representativeness of the participants of the ROAD baseline study to the Japanese population by comparing anthropometric measurements and frequencies of smoking and alcohol consumption between participants and the general Japanese population [5]. Values for the general population were obtained from the 2005 National Health and Nutrition Survey conducted by the Ministry of Health, Labour and Welfare in Japan [20]. Regarding anthropometric measurements, we found no significant differences between our participants and the total Japanese population, except that men participants aged 70–74 years in the ROAD study were significantly smaller in terms of body structure than the overall population (*p* < 0.05). In addition, proportions of current smokers and current drinkers were significantly higher in the general Japanese population than in the study population [5], suggesting that participants of the ROAD study have healthier lifestyles than the general population. This “healthy” selection bias should be taken into consideration when using reference values obtained in the ROAD study.

In conclusion, we have assessed whether the proposed clinical decision limits of the indices in locomotive syndrome risk test stages 1 and 2 could predict immobility represented by a slow FTSST time and slow walking speed using data from a population-based cohort of the third survey of the ROAD study. We found that all the indices in the locomotive syndrome risk test—the two-step test, stand-up test, and 25-question GLFS—could significantly and independently predict a decline in mobility and that the accumulation of such indices increased the risk of immobility exponentially.

## Electronic supplementary material

Supplementary material 1 (DOCX 11 kb)
