# Supplementary material for: Association between new indices in the locomotive syndrome risk test and decline in mobility: third survey of the ROAD study
Source: J Orthop Sci. 2015 Jun 25;20(5):896–905. doi: 10.1007/s00776-015-0741-5 (PMC4575347; doi:10.1007/s00776-015-0741-5)
Supplement: Supplementary file 1 — Supplementary material 1 (DOCX 11 kb) [file 776_2015_741_MOESM1_ESM.docx]

**Supplementary Table 1. Comparison between results of the 25-question geriatric locomotive function scale (GLFS) and the five-question GLFS**

|  | 25-question GLFS score | | |
| --- | --- | --- | --- |
| Five-question GLFS score | ≥16 | <16 | Total |
| ≥6 | 140 | 21 | 161 |
| <6 | 23 | 1351 | 1374 |
| Total | 163 | 1372 | 1535 |

Sensitivity = 140/163 = 85.9%

Specificity = 1351/1372 = 98.5%
